# Alleviation of Postharvest Chilling Injury of Carambola Fruit by γ-aminobutyric Acid: Physiological, Biochemical, and Structural Characterization

**DOI:** 10.3389/fnut.2021.752583

**Published:** 2021-11-16

**Authors:** Francine Ngaffo Mekontso, Wenhui Duan, El Hadji Malick Cisse, Tianye Chen, Xiangbin Xu

**Affiliations:** ^1^College of Food Science and Engineering, Hainan University, Haikou, China; ^2^School of Life Science, Hainan University, Haikou, China; ^3^Key Laboratory of Food Nutrition and Functional Food of Hainan Province, Haikou, China

**Keywords:** carambola fruit, γ-aminobutyric acid, chilling injury, redox-homeostasis, fatty acids

## Abstract

Chilling injury is a physiological disorder affecting the quality of carambola fruit. In the present study, the effect of exogenous γ-aminobutyric acid (GABA) on CI development in carambola fruit during storage at 4°C for 15 days was investigated. The results showed that 2.5-mM GABA reduced CI index, maintained pericarp lightness, and decreased the electrolyte leakage (EL) and malondialdehyde content (MDA) while increased the superoxide dismutase (SOD), peroxidase (POD), and catalase (CAT) enzyme activities. Endogenous GABA content was significantly higher in the treated fruit than in the control fruit during the whole storage. Besides, the treatment promoted the accumulation of proline and ascorbic acid (AsA) under chilling stress. Compared to the control, GABA-treated fruit exhibited a higher activity of phenylalanine ammonia-lyase (PAL) and total phenolic compounds, and a lower activity of polyphenol oxidase (PPO). In addition, the Safranin O/fast green staining revealed *via* microscopic images that the GABA treatment reduced the cell walls degradation of carambola fruit. Moreover, the results displayed a lower activity of phospholipase D (PLD) and lipoxygenase (LOX) enzymes, which coincided with a higher content of oleic acid (C18:1), linoleic acid (C18:2n6), and α-linolenic acid (C18:3n3) after 15 days of treatment, leading to the maintenance of the integrity and prevention of the membrane of the rapid softening of carambola fruit. The findings of the present work showed particularly new insights into the crosstalk between GABA and fatty acids. GABA might preserve the pericarp of carambola fruit by increasing the content of the unsaturated fatty acid (UFA) γ-linolenic acid and reducing the saturated fatty acid (SFA) such as caproic acid (C6:0), caprylic acid (C8:0), myristic acid (C14:0), and palmitic acid (C16:0) progressively. GABA can be used as an appropriate postharvest technology for improving the quality of carambola fruit during low-temperature storage.

## Introduction

Carambola (*Averrhoa carambola*) fruit, also called “star fruit” is a non-climacteric fruit mainly cultivated in subtropical and tropical regions such as Southeast Asia and America (1, 2). It is one of the most popular fruit among consumers because of its succulent pulp, attractive flesh, distinctive flavor, and unique taste. The fruit is an important source of vitamins, minerals, dietary fiber, and essential antioxidants, which are beneficial for human health (3–5). Since carambola fruit ripens and rapidly spoils when placed at ambient temperature, cold storage is a commonly technology used to extend postharvest shelf life and maintain fruit quality (5, 6). However, carambola fruit is highly susceptible to cold storage, developing chilling injury (CI) symptoms, and suffering weight and firmness loss at long exposure to temperature above 2°C and below 5°C (5, 7). Cold stress is one of the critical environmental factors that cause several damages and abnormalities in horticultural plants species (8). Cold can trigger oxidative stress in a plant at the subcellular compartments by increasing the production of reactive oxygen species (ROS), such as singlet oxygen (O_2_), superoxide radicals (${\text{O}}_{2}^{.-}$), hydrogen peroxide (H_2_O_2_), and hydroxyl free radicals (.OH) (9). The regulation of the ROS pathway may reduce the deleterious effects of environmental stressors and consequently contribute to improving fruit quality (9, 10). Therefore, antioxidant enzymes have been found to play an essential role in protecting cell membranes from oxidative stress and repairing membrane lipid by scavenging ROS, thereby enhancing chilling tolerance (11–13). Low temperature can also cause the dysfunction of membrane structure, leading to the change in lipid composition, thus developing CI symptoms (14). Since plant membrane is considered as a protective barrier against abiotic stress, substantial evidence has shown that the phase transition in cell membrane lipid from liquid-crystalline to the solid-gel state under chilling stress leads to decreased membrane permeability and promoted CI occurrence (15, 16). The membrane lipid peroxidation induced by chilling temperature triggered the activity of phospholipase D (PLD) and lipoxygenase (LOX), which catalyzed the peroxidation of membrane unsaturated fatty acids (UFA), leading to the loss of membrane fluidity assayed by increased electrolyte leakage (EL) and malondialdehyde (MDA) content (10, 17). Recently, Gao et al. (12) have shown delayed CI in peach fruit-inhibited lipid peroxidation and increased fatty acids unsaturation, mainly linoleic acid (C18:2) and linolenic acid (C18:3). The greater degree of membrane lipid unsaturation lowers the phase transition temperature. The main symptoms of CI in carambola fruit include dark green-brown pitting surface, shriveling, dark rib-edge, discoloration, and failure to ripen properly, which tend to aggravate when the fruit is brusquely transferred to 25°C (5). Owing to the high amount of perishability of carambola fruit, the commercial losses issues faced by sellers during transportation over long distance, and the great demand in the global market, it is indispensable and urgent to develop appropriate treatments to diminish its CI. Therefore, researchers have made great efforts to deal with this problem by developing postharvest techniques such as methyl jasmonate (3), polyamines (4), modified atmosphere packaging (18), and salicylic acid (19) treatments.

The γ-aminobutyric acid (GABA) is a ubiquitous non-proteogenic amino acid found in bacteria, animals, and plants (20). GABA biosynthesis is formed by a short metabolic pathway called GABA shunt that consists of three enzymes, such as glutamate decarboxylase (GAD), GABA transaminase (GABA-T), and succinic semialdehyde dehydrogenase (SSADH) (20–23). GABA is considered as a natural signaling molecule with the ability to modulate the antioxidant system in response to biotic and abiotic stresses (23). In higher plants, GABA is synthesized in low concentration at normal conditions but can be rapidly enhanced in response to various stressful conditions (20, 23). Recently, Shekari et al. (24) have reported that exogenous application of GABA enhanced the endogenous GABA content and retarded cap browning of button mushrooms (*Agaricus bisporus*) by increasing the expression of the *GAD* gene and decreasing the expression of the GABA transaminase gene. The effect of GABA treatment on reducing CI of peach fruit might be due to its capability to enhance the accumulation of endogenous GABA and proline content, which resulted from the increased GAD, pyrroline-5-carboxylate synthetase (P5CS), and ornithine δ-aminotransferase (OAT) activities, and decreased proline dehydrogenase (PDH) activity (25). In cucumber fruit, GABA treatment improves the antioxidant machinery and decreases the level of ROS, which is associated with the induction of CI tolerance (10). GABA treatment could be applied to delay the development of CI symptoms of anthurium cut flowers by enhancing the activity of the GABA shunt to produce energy (15). The mitigation of CI by GABA treatment could improve cold tolerance in citrus fruit and zucchini fruit *via* the GABA-shunt pathway (21, 22). In addition, Aghdam et al., (26) has shown that the application of GABA treatment in anthurium cut flowers inhibited PLD and LOX activity and enhanced UFA/saturated fatty acids (SFA) ratio, which results in the improvement of chilling tolerance. He et al. (17) reported that there is little knowledge about the physiological and biochemical characteristics of biomembrane fruit during storage.

Mounting evidence has shown the crucial role of GABA against CI in various fruits (24, 27), but the influence and interactions of exogenous GABA with the fatty acids and antioxidant machinery remained elusive. To date, no study has focused on the effect of exogenous GABA in carambola fruit. Therefore, this study aimed to evaluate how GABA could boost the chilling tolerance of carambola fruit through the evaluation of the antioxidant defense system and the fatty acids composition.

## Materials and Methods

### Plant Materials and Postharvest Treatments

Carambola (*A. carambola* cv. Xiangmi) fruit was harvested at the mature dark green stage from a local orchard in Haikou City, Hainan Province, P.R. China. The fruits with uniform shape and color without any physical damages were selected and then immediately transported to the laboratory. All fruits randomly divided into two lots of 180 fruits each were thoroughly cleaned with tap water and then air-dried at ambient temperature prior to use. The first lot of fruit was dipped in 2.5-mM GABA solution for 5 min, whereas the second lot was immersed in sterile deionized water for 5 min (as control). The above GABA concentration was selected as optimal according to the preliminary experiments, utilizing 1, 2.5, 5, 10, and 20 mM (Supplementary Figure 1). Afterward, the fruit was air-dried for approximately 30 min and stored for 15 days at 4°C and 85-90% relative humidity. Samples were collected from 12 fruits of each treatment on 5-day intervals for 15 days, and then removed after 10 and 15 days of storage at 4°C and held at 25°C to simulate shelf conditions. These collected samples were immediately frozen in liquid nitrogen and kept at −80°C. Each treatment was repeated three times, and the entire experiment was repeated two times.

### Determination of CI Index and Color Changes

CI was performed according to the method described by Pérez-Tello et al. (7). The degree of CI was visually evaluated by rating the browning severity on the surface area of the peel fruit and was divided into four classes: 0 = no CI; 1= slight injury (surface browning < 10%); 2 = moderate injury (10–20% of surface browning); 3 = severe injury (more than 20% of surface browning). CI index was calculated using the following formula: CI index = ∑ [(chilling scale) × (number of fruit at that chilling scale)]/(3 × total number of fruits in each treatment) × 100.

The color was determined using CR-400 Minolta Chroma Meter (Minolta Camera Co. Ltd, Osaka, Japan), which provides CIE L^*^ (lightness or darkness), a^*^ (green to red), and b^*^ (blue to yellow) values. Ten fruits per treatment were examined. The skin color was longitudinally measured on three different locations (proximal end, middle and distal ends of the ribs) of each fruit.

### Determination of Weight Loss, Respiration, and Firmness

To determine the weight loss, both control and treated fruits were weighed at intervals using a digital balance (Mettler-Toledo Instruments Inc., Shanghai, China). Weight loss was calculated as W_0_ - W_f_/W_0_ × 100, where W_0_ is the initial weight, and W_f_ is the final weight. Weight loss was expressed as a percentage (%) of fresh weight. The respiration rate was monitored using a portable infrared CO_2_ gas analyzer (GXH-3010E, Beijing Huayun Analytical Instrument Research Institute Co. Ltd., China). Six fruits per replicate of each treatment were sealed in 2.25-L glass jars at 25°C for.5 h. The respiration rate was expressed as mg kg^−1^ h^−1^.

The firmness of pulp was measured at the equatorial zone of each carambola fruit (six fruits per replicate) using TA. XT. Plus Texture Analyser (Stable Micro Systems, Godalming, Surrey, UK) equipped with a 5-mm diameter probe at a speed of 5 mm s^−1^. The reading was expressed in Newtons (N) as the mean of five measurements recorded at five locations along with one of the fruit ribs.

### Determination of Enzyme Activities

The enzyme activities of superoxide dismutase (SOD), peroxidase (POD), catalase (CAT), phenylalanine ammonia-lyase (PAL), and polyphenol oxidase (PPO) were determined following the manufacturer's Solarbio Assay Kit instructions (Solarbio Inc., Beijing, China). In brief, carambola pericarp tissues were removed from −80°C and then immediately grounded finely in liquid nitrogen. The frozen powder was weighed (0.1 g) and homogenized in 1 ml of the liquid solution. The SOD, POD, CAT, and PPO enzymes were centrifuged at 8,000 × g for 10 min at 4°C. The PAL enzyme was centrifuged at 10,000 × g for 10 min at 4°C. Afterwards, the supernatants were collected and then used for studying enzyme activity. The absorbance of SOD, POD, CAT, PAL, and PPO enzyme activities was measured by a 96-well microplate reader (SpectraMax190; Molecular Devices, Sunnyvale, CA, USA), using a UV spectrophotometer at 560, 470, 240, 290, and 410 nm, respectively, and all enzymes were expressed as U g^−1^.

### Determination of Ascorbic Acid (AsA), Total Phenol and Proline Contents, EL and MDA Content

The AsA content was determined following the manufacturer's ELISA KIT instructions (Jining Industrial Inc., Shanghai, China). Briefly, peel tissues were grounded with liquid nitrogen, and 0.1 g was weighed and homogenized with 0.9 ml of phosphate-buffered saline (PBS). The supernatant was collected after centrifugation at 3,000 × g for 20 min. The absorbance was measured at 450 nm. AsA content was calculated using the standard curve and expressed as μmol L^−1^. Total phenol content was determined according to the Folin–Ciocalteu reagent described by Hinneburg et al. (28). The absorbance of total phenol content was measured at 765 nm, and the results were expressed as mg of gallic acid equivalent per 100 g on a fresh-weight basis. The proline content was conducted according to Gao et al. (29), with some alterations. The absorbance of the reaction mixture was measured at 520 nm. Proline content was calculated using the standard curve and expressed as μg g^−1^.

The EL was measured using the method described by Liu et al. (30), with some alterations. Carambola tissues were cut with a 1-cm-diameter stainless steel cork borer from the equatorial zone into round disk and washed two times with deionized water. After being dried on absorbent paper, the disk were immediately placed into a beaker containing 40 ml of deionized water and shaken on a laboratory plate shaker for 1 h at 25°C. The conductivity (L_0_) was measured using a conductivity meter (Mettler-Toledo Instruments Inc., Shanghai, China) as the initial EL, and (L_1_) was measured 5 min afterward. The total electrical conductivity was obtained by boiling the sample for 20 min, and then cooled at ambient temperature. The conductivity (L_2_) was measured. EL was calculated as the following formula: [(L_1_ - L_0_)/(L_2_ - L_0_)] × 100 and expressed as a percentage (%) of the total conductivity. The MDA content was determined according to the method described by Liu et al. (30), with some modifications. The optical density (OD) was measured at 532, 450, and 600 nm using a UV spectrophotometer. The MDA content was calculated according to the formula: 6.45 × (OD_532_ – OD_600_) −0.56 × OD_450_ and expressed as μmol g^−1^ FW.

### Tissue Staining Analysis

Tissues collected from both control and treated fruit on 0, 10, 15, 10 + 9, and 15 + 6 days were cut using a sharp razor blade with approximate dimensions of 3 × 3 mm (length × width). The samples were immediately kept into a tube containing 20 ml of fixative solution FAA [90% ethanol (v/v), 5% formalin (v/v), 5% acetic acid (v/v)] for at least 24 h. The experiment was conducted according to the protocol described by Li et al. (31), and each tissue staining was replicated three times.

### Determination of PLD and LOX Enzyme Activities

PLD enzyme was measured by an enzyme-linked immunosorbent assay (ELISA). The Plant phospholipase D (PLD) ELISA Kit was used following the manufacturer's instructions (Jiangsu Mei Biao Biological Technology Co., Ltd, Jiangsu, China). Briefly, carambola pericarp tissues were removed from −80°C and then immediately grounded finely in liquid nitrogen. The frozen powder was weighed (0.1 g) and homogenized in.9 ml of the phosphate-buffered saline (PBS, pH 7.4). After centrifugation at 3,000 × g for 20 min at 4°C, the supernatant was collected and used for the measurement of PLD activity. The absorbance of PLD enzyme activity was measured by a 96-well microplate reader (SpectraMax190; Molecular Devices, Sunnyvale, CA, USA) using a UV spectrophotometer at 450 nm and expressed as U g^−1^.

LOX enzyme was determined following the manufacturer's Solarbio Assay Kit instructions (Solarbio Inc., Beijing, China). Briefly, carambola pericarp tissues were removed from −80°C and then immediately grounded finely in liquid nitrogen. The frozen powder was weighed (0.1 g) and homogenized in 1 ml of the liquid solution. After centrifugation at 16,000 × g for 20 min at 4°C, the supernatant was collected and used as crude enzyme extract. The absorbance of LOX enzyme activity was measured by a 96-well microplate reader (SpectraMax190; Molecular Devices, Sunnyvale, CA, USA) using a UV spectrophotometer at 234 nm and expressed as U g^−1^.

### Determination of Endogenous GABA Content

Endogenous GABA content was determined following the manufacturer's Plant γ-aminobutyric acid (GABA) Assay Kit instructions (Suzhou Comin Biotechnology Co., Ltd, Suzhou, China), and the GABA content was expressed as mg g^−1^.

### Gas Chromatography (GC) Analysis of Fatty Acid

Carambola peel collected from 0, 10, 15, 10 + 9, and 15 + 6 days were used for the GC analysis of fatty acid. Briefly, frozen samples were homogenized in 100-μL internal standard (heptadecanoic acid), then 2 ml of 5% concentrated sulfuric acid (H_2_SO_4_)/methanol solution and 300 μL of toluene were added into the bottle. The headspace of the bottle was sealed tightly with an aluminum cap with a polytetrafluoroethylene (PTFE) gasket, vortexed with a vortex 16700 Mixer Thermolyne (Maxi-Mix 1, Lowa, US), heated in a water bath W350 Memmert (Memmert GmbH and Co, Schwabach, Germany) for 1 h at 95°C and then cooled at room temperature (25°C). Furthermore, 2 ml of.9% NaCl solution was added, and the mix solution was slightly shaken. Extracting solvent, 1 ml of n-hexane was added, and then the mix was centrifuged at 5,000 rpm for 5 min. The supernatant was transferred into a vial. The GC analyses were performed on an Agilent 7890A gas chromatography system (Agilent Technologies, California, US), equipped with a hydrogen flame ionization detector (FID) and an Agilent J&W DB-Fast FAME GC columns with the splitless injector (split ratio, 20:1). Injector and detector temperatures were set at 250 and 260°C, respectively. The GC oven conditions were conducted as follows: 80°C (hold.5 min), to 165°C at 40°C/min (hold 1 min), to 230°C at 4°C/min (hold, 6 min).

### Statistical Analysis

Experiments were carried out using a completely randomized design. Data were analyzed by two-way ANOVA with SPSS 20.0 (SPSS, Chicago, IL, USA). Statistically significant differences were performed by Tukey's HSD (honestly significant difference) at ^*^p < 0.05, ^**^p < 0.01, ^***^p < 0.001, ^****^p < 0.0001. The results were expressed as mean ± standard deviation (SD), and Graph Pad Prism 9.0.0 software was used to draw the graphs and analyze the data.

## Results

### CI Index and Color Changes

**Figure 1:**
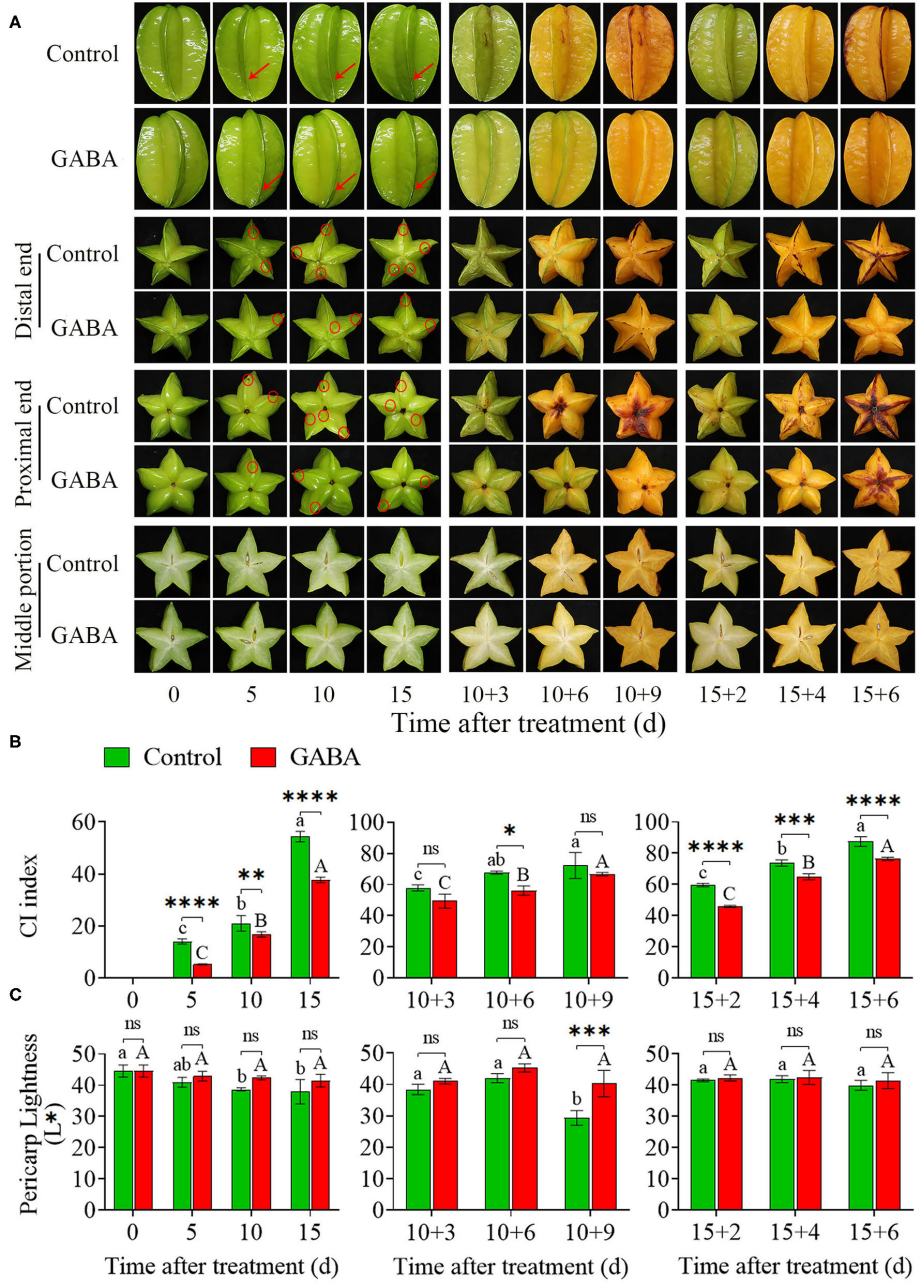
Visual appearance of CI symptoms of carambola fruit during storage at 4 and 25°C **(A)**, CI index **(B)**, and pericarp lightness **(C)**. Error bars are expressed as means ± SD (standard deviation) of three replicates (*n* = 3). The asterisks indicate statistically significant differences between treatments at ^*^p < 0.05, ^**^p < 0.01, ^***^p < 0.001, ^****^p < 0.0001, ns, no significant.

The GABA treatment could effectively retard the appearance of the ribs-edge browning, a typical CI symptom in carambola fruit under cold stress (Figure 1). GABA-treated fruit did not exhibit any CI symptoms, whereas early symptoms have started to be visible in the control fruit from 5 to 15 days of storage at 4°C. Thus, these damages substantially developed and became more severe with time, especially when the fruits were transferred at 25°C (Figure 1A). Besides, the CI index in both control and GABA-treated fruits increased gradually with storage time, but the application of GABA treatment remarkably inhibited the increase of CI index in carambola fruit (Figure 1B). In comparison with the control, carambola fruit treated with GABA displayed lower CI index from 0 to 15 days during storage at 4°C. Moreover, from 10 + 3 to 10 + 9 days at 25°C, the CI index in the control fruit continually increased from 57.89 to 72.28, while it was reduced in treated fruit from 49.45 to 66.67. A similar increase was obtained from 15 + 2 to 15 + 6 days of storage at 25°C in which CI index was consistently higher in the control than in the treated fruit (Figure 1B). The color changes generally reflected the rapid development of CI in the pericarp of the fruit. As shown in Figure 1C, chilling stress induced color changes in carambola fruit, and the pericarp lightness expressed as L^*^ value declined gradually with the increase of storage duration. The GABA treatment maintained the higher L^*^ value in comparison with the control fruit. From 0 to 15 days at 4°C, the L^*^ value in control fruit decreased sharply from 44.60 to 37.93, while it dropped slowly from 44.60 to 41.52 in GABA-treated fruit (Figure 1B). A similar result was found from 10 + 3 to 10 + 9 days and 15 + 2 to 15 + 6 days at 25°C in which the lightness decreased slightly in treated fruit compared with the control fruit (Figure 1C). These results indicated that GABA treatment could positively affect the alleviating CI of carambola fruit during cold storage.

### Weight Loss, Respiration Rate, and Firmness

**Figure 2:**
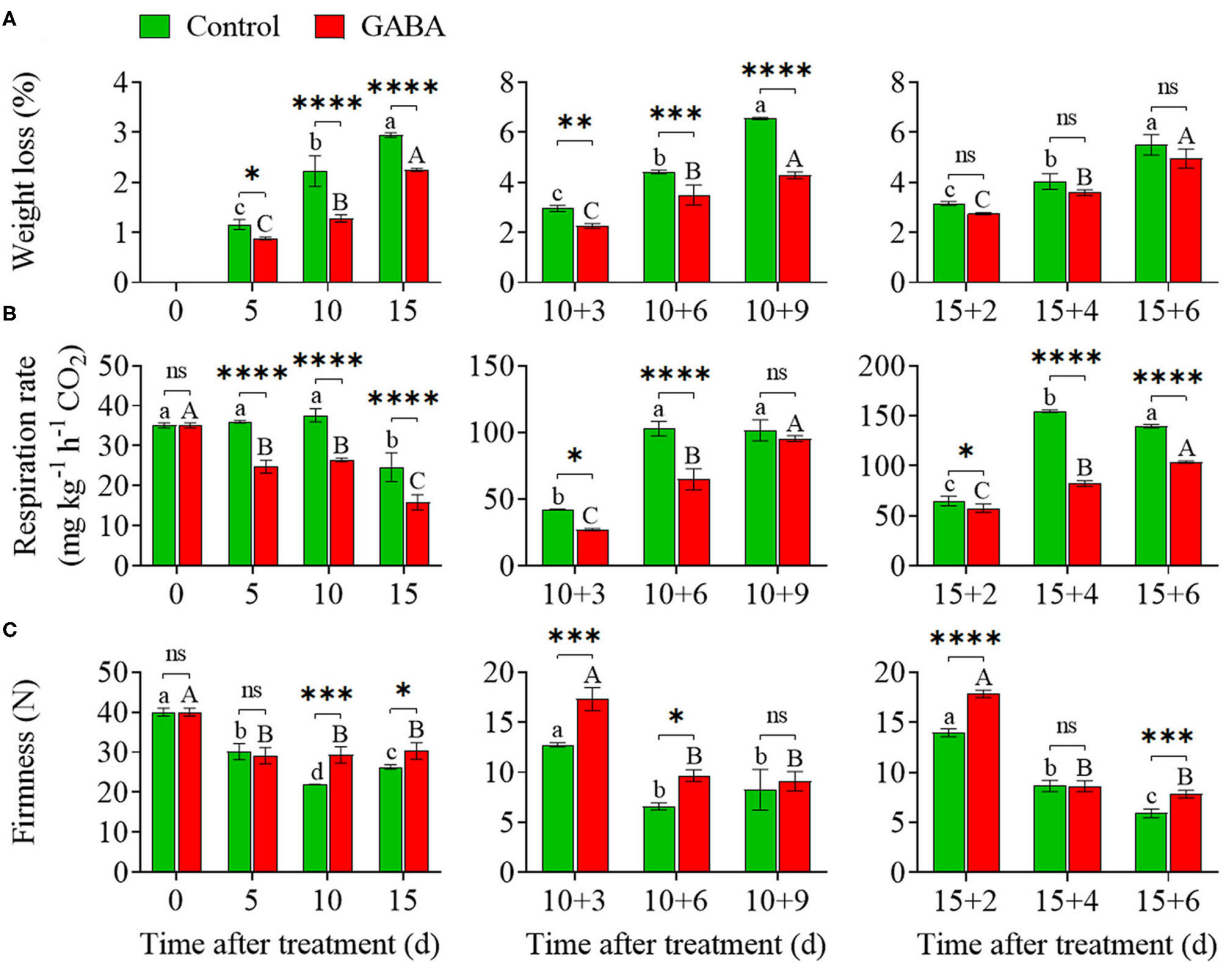
The weight loss **(A)**, respiration rate **(B)**, and firmness **(C)** of carambola fruit during storage at 4 and 25°C. Error bars are expressed as means ± SD (standard deviation) of three replicates (*n* = 3). The asterisks indicate statistically significant differences between treatments at ^*^p < 0.05, ^**^p < 0.01, ^***^p < 0.001, ^****^p < 0.0001, ns, not significant.

Weight loss increased steadily throughout the storage time. In general, comparable weight losses were noticed in control and GABA-treated fruit during chilling stress. The application of GABA treatment markedly reduced the weight loss within the range of 0 to 2.25% compared with the control (range of 0–2.94%) from 0 to 15 days at 4°C (Figure 2A). On 10 + 9 days at 25°C, the weight loss of control fruit increased considerably to a maximum value of 6.55%, and the treated fruit reached 4.28% (Figure 2A). Also, on 15 + 6 days at 25°C, weight loss was slower in GABA-treated fruit than in control. As shown in Figure 2B, the application of GABA treatment on carambola fruit provoked a noticeable decrease in the respiration rate. After 15 days of exposure to low temperature, the treated fruit exhibited a low respiration rate of 15.86 *vs*. 24.68-mg kg^−1^ h^−1^ CO_2_ in control, which subsequently increased after 10 + 9 and 15 + 6 days (99.19 and 117.73-mg kg^−1^ h^−1^ CO_2_, respectively) when transferred to ambient temperature but was still lower than that in the control fruit (103.71- and 139.40-mg kg^−1^ h^−1^ CO_2_, correspondingly) (Figure 2B). Overall, a lower value of the respiration rate was observed for the treated fruit over the storage period.

Fruit firmness slowly declined during a long exposure at low temperature. The firmness in control decreased within a range of 26.29 N after 15 days of storage at 4°C and after 10 + 9 and 15 + 6 days (8.25 and 5.90 N, respectively) of storage at 25°C (Figure 2C). Meanwhile, firmness in fruit treated with exogenous GABA remained relatively steady with a value of 30.31 N after 15 days of storage at 4°C and after 10 + 9 and 15 + 6 days (9.11 and 7.83 N, respectively) of storage at 25°C (Figure 2C). Nevertheless, treated fruit showed a further reduction to 29.15 N compared with control, which was 30.12 N after 5 days of the storage period (Figure 2C).

### SOD, CAT, POD, PAL, and PPO Enzyme Activities

**Figure 3:**
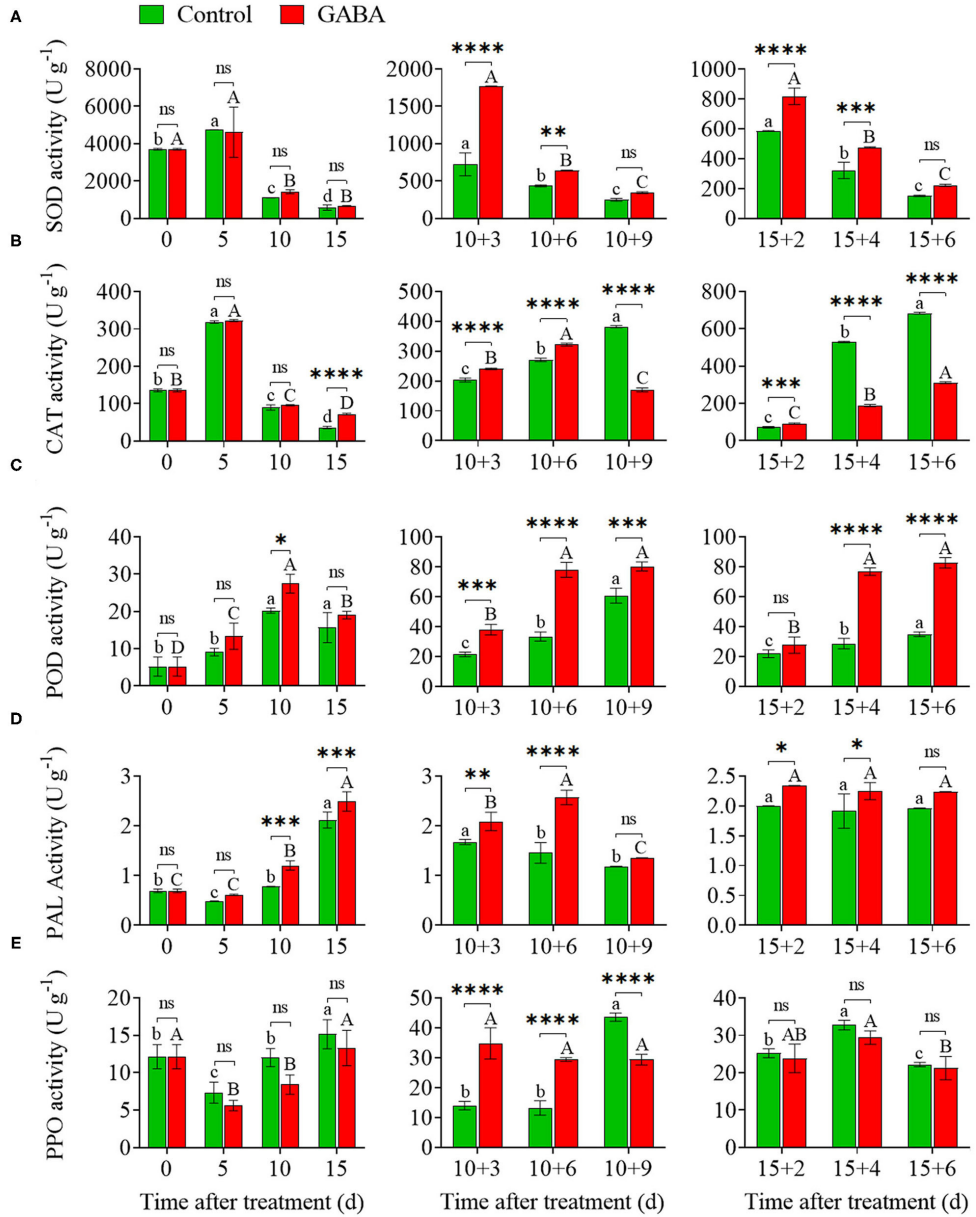
The superoxide dismutase (SOD) **(A)**, catalase (CAT) **(B)**, peroxidase (POD) **(C)**, phenylalanine ammonia-lyase (PAL) **(D)**, and polyphenol oxidase (PPO) **(E)** activities of carambola fruit during storage at 4 and 25°C. Error bars are expressed as means ± SD (standard deviation) of three replicates (*n* = 3). The asterisks indicate statistically significant differences between treatments at ^*^p < 0.05, ^**^p < 0.01, ^***^p < 0.001, ^****^p < 0.0001, ns, not significant.

The GABA treatment enhanced SOD, CAT, POD, PAL activities and minimized PPO activity in carambola fruit compared with control under chilling stress (Figure 3). SOD and CAT activities in treated fruit were increased on 5 days at 4°C, reaching a peak value of 4,743.71 and 4,616.83 U g^−1^, respectively, and then dramatically decreased to a level almost similar to that of the control at the end of storage at 4°C (Figures 3A,B). However, SOD activity was lower in treated fruit than that in control on 5 days of cold storage (Figure 3A). GABA-treated fruit exhibited an increase in SOD activity, reaching a peak on 10 + 3 and 15 + 2 days at 25°C compared with the control, and then decreased afterward (Figure 3A). The SOD activity in GABA-treated and control fruit was 1,768.61 and 721.82 U g^−1^ on 10 + 3 days, whereas it was 344.03 and 249.02 U g^−1^ on 10 + 9 days, respectively. Also, SOD activity in treated and control fruit was 818.68 and 586.10 U g^−1^ on 15 + 2 days, whereas it was 222.15 and 152.55 U g^−1^ on 15 + 6 days, respectively. Meanwhile, CAT activity was affected by the non-chilling temperature. CAT activity was obviously elevated in GABA-treated fruit on 10 + 3, 10 + 6, and 15 + 2 days, then dropped sharply on 10 + 9, 15 + 4, and 15 + 6 days compared with the control fruit (Figure 3B). As shown in Figures 3C,D, POD and PAL activities in treated fruit increased transiently to the above-the-control level after 15 days of chilling stress, reaching a value of 19.00 and 2.49 U g^−1^, respectively. However, once GABA-treated fruits were transferred to 25°C, a rapid increase in the POD and PAL level occurred, reaching values even higher than those found in control (Figures 3C,D).

PPO activity in both control and GABA-treated fruit slightly decreased during the first 5 days and then gradually increased to a value of 15.13 and 13.30 U g^−1^on 15 days at 4°C, respectively (Figure 3E). Thereafter, PPO activity in the control fruit firstly declined from 10 + 3 to 10 + 6 days compared with the treated fruit, and then enhanced considerably with a value of 43.61 U g^−1^ on 10 + 9 days. The value of PPO activity in the control fruit remained high from 15 + 2 to 15 + 6 days, reaching a peak of 32.77 U g^−1^ on 15 + 4 days at 25°C. However, GABA-treated fruit displayed a similar pattern but at a lower PPO activity than the control fruit during the entire storage (Figure 3E).

### The Content of AsA, Total Phenol, and Proline, EL, and MDA Content

**Figure 4:**
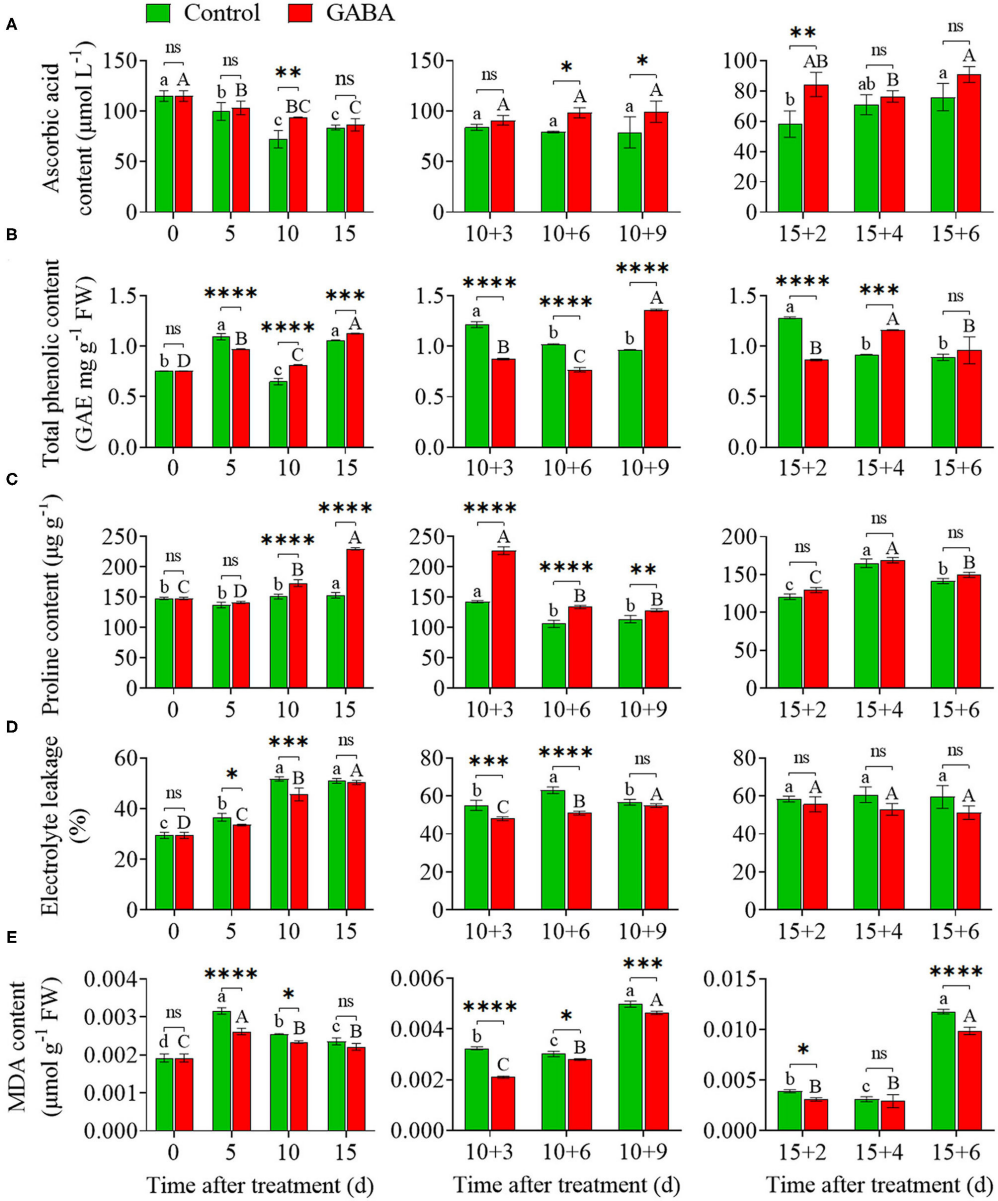
The content of ascorbic acid **(A)**, total phenol **(B)**, and proline **(C)**, electrolyte leakage **(D)**, and malondialdehyde content (MDA) content **(E)** of carambola fruit during storage at 4 and 25°C. Error bars are expressed as means ± SD (standard deviation) of three replicates (*n* = 3). The asterisks indicate statistically significant differences between treatments at ^*^p < 0.05, ^**^p < 0.01, ^***^p < 0.001, ^****^p < 0.0001, ns, not significant.

The increases of AsA and proline concentrations were recorded in treated fruit, which were considerably higher than those in control fruit after 15 days under cold storage (Figures 4A,C). The relative AsA and proline content in treated fruit was 86.49 μmolL^−1^ and 229.92 U g^−1^, respectively, while it was 83.77 μmolL^−1^ and 153.35 U g^−1^in control fruit, respectively, on 15 days at 4°C. Unlike AsA and proline, total phenol content first dropped to the below-the-control level at 5 days and then increased after 15 days (Figure 4B). GABA treatment maintained the higher content of AsA and proline in GABA-treated fruit than that in control after the fruits were transferred to 25°C (Figures 4A,C). Meanwhile, the content of total phenol in carambola fruit was affected by the treatment under non-chilling temperature. Total phenol was lower in treated fruit during 10 + 3, 10 + 6, and 15 + 2 days at ambient temperature. However, it was strongly higher on 10 + 9, 15 + 4, and 15 + 6 days than that in control fruit (Figure 4B).

As shown in Figures 4D,E, both EL and MDA content in control and treated fruit displayed an upward trend, which was often associated with the membrane deterioration of carambola fruit under chilling stress. Therefore, compared with the control, treated fruit showed lower EL and MDA accumulation during the entire storage at 4°C. A substantial increase in EL appeared after 5 days of cold storage, and the control fruit had a higher EL than that in the treated fruit (Figure 4D). MDA content in control fruit first climbed up on 5 days then decreased on 10 and 15 days under chilling stress (Figure 4E). Thereafter, MDA content and EL continually increased at ambient temperature, but GABA treatment suppressed the increases of MDA in treated fruits that were stored anywhere from 10 + 3 to 10 + 9 days and 15 + 2 to 15 + 6 days (Figure 4E). It also inhibited the increases of EL in treated fruit stored at the same storage at 25°C (Figure 4D).

### The Epidermal Cells Observation

**Figure 5:**
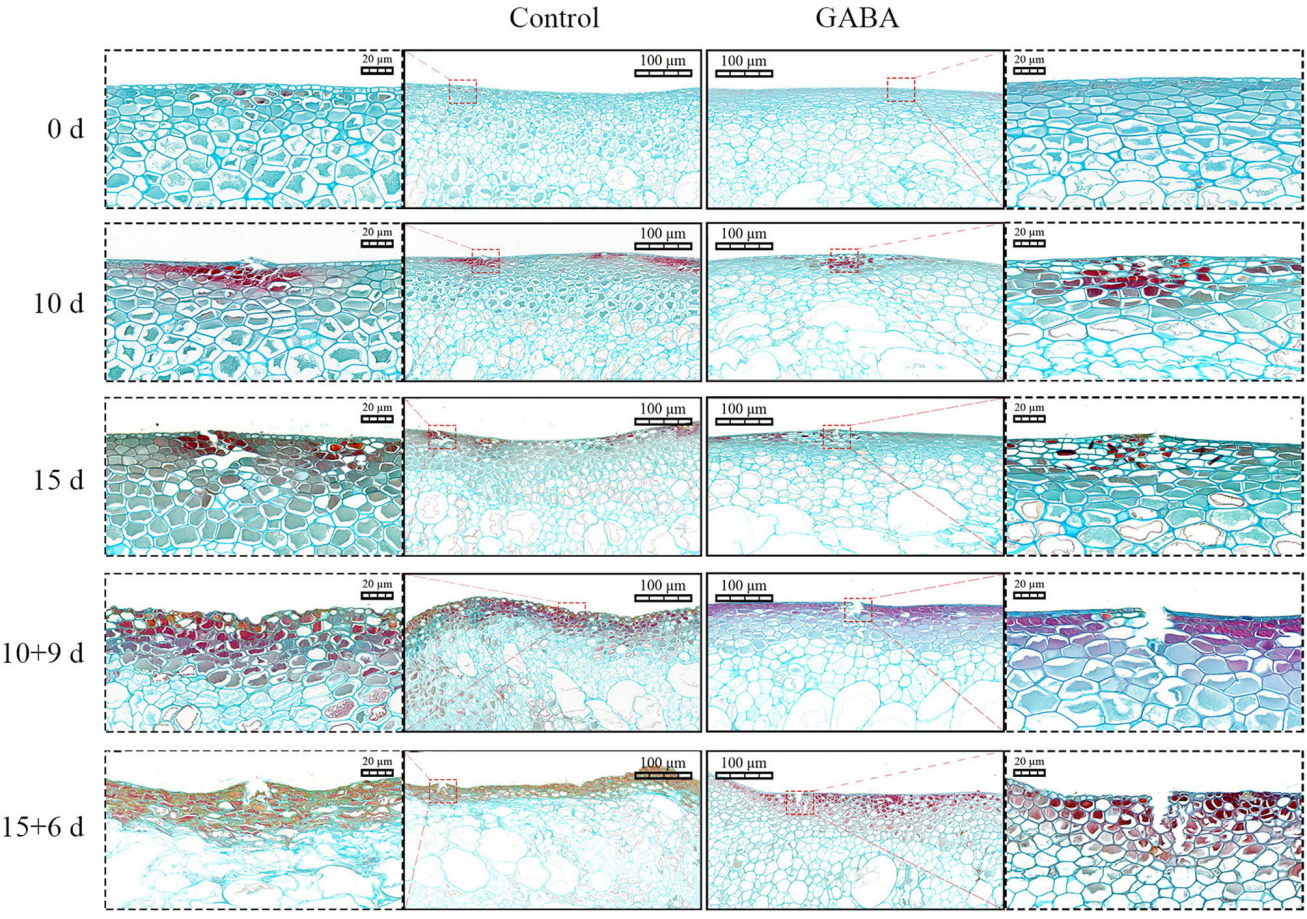
The light microscopy images of a transverse section stained with safranin O/fast green of carambola fruit tissues stored at 4 and 25°C. The red dotted squares indicate the injured cell wall. Scale bars = 20 and 100 μm.

Staining analysis was performed using safranin O/fast green to visualize changes caused by chilling stress in epidermal cells and highlight the effect of GABA treatment on CI at the epidermal sites during storage at 4 and 25°C. Safranin O/fast green staining revealed that the cell walls of control fruit were more damaged than those in treated fruit. Light microscopy observations showed no sign of cell damage or disruption in the transverse section of both control and treated fruits at 0 day of storage (Figure 5). However, injured cell walls had started to be visible on 10 days at 4°C and became more severe in control than in treated fruit on 10 + 9 and 15 + 6 days at 25°C. Thus, GABA-treated fruit displayed less severe crack across the cell walls from 10 to 15 + 6 days. In contrast, control fruit exhibited severe crack (10 and 15 days at 4°C) with flattened and deformed cell walls on 10 + 9 and 15 + 6 days at 25°C. These results indicated that GABA treatment reduced chilling damages and maintained cell wall integrity in treated fruit compared with control (Figure 5).

### PLD and LOX Enzyme Activities

**Figure 6:**
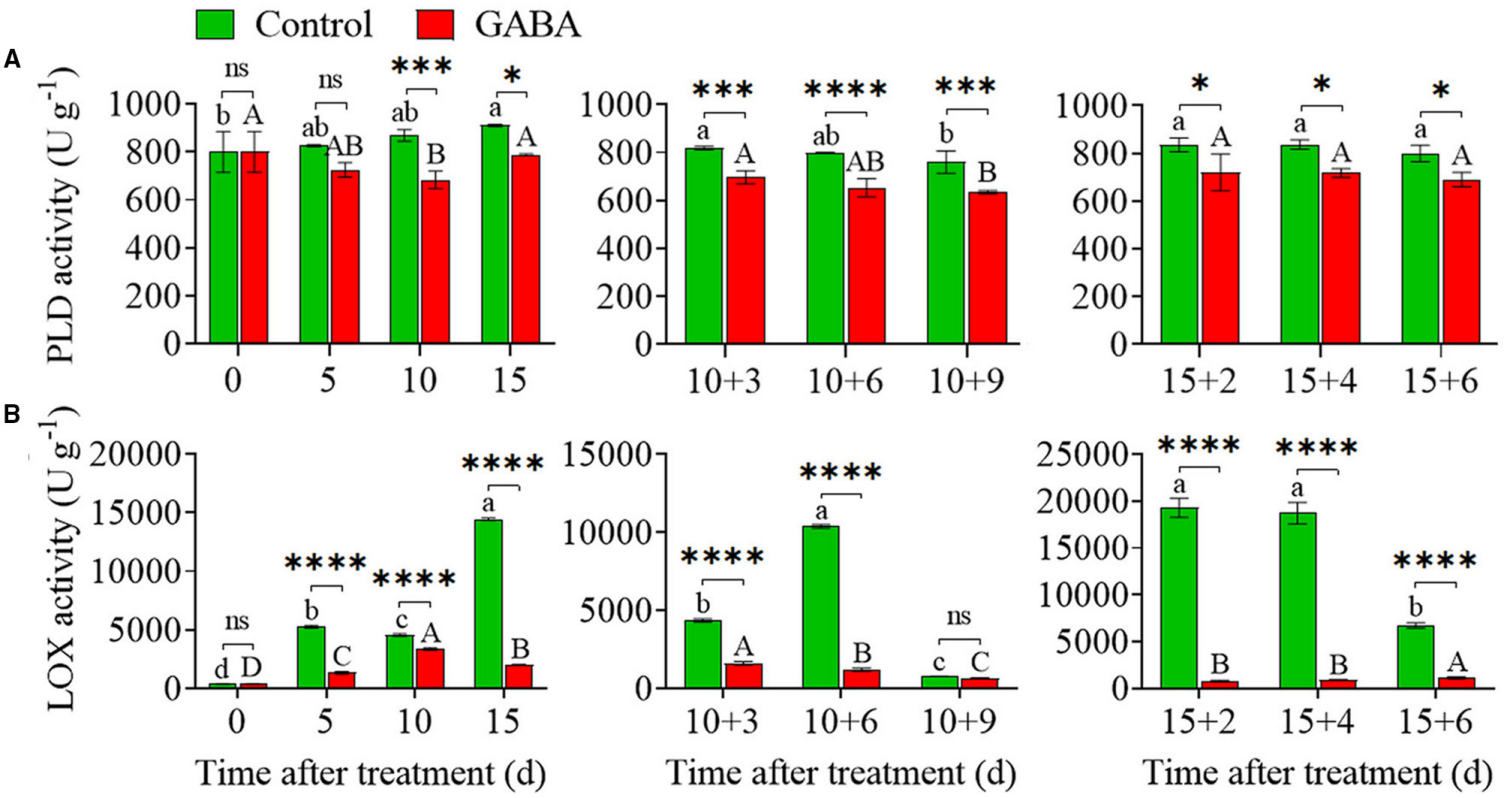
The phospholipase D (PLD) **(A)** and lipoxygenase (LOX) **(B)** activities of carambola fruit during storage at 4 and 25°C. Error bars are expressed as means ± SD (standard deviation) of three replicates (n = 3). The asterisks indicate statistically significant differences between treatments at ^*^p < 0.05, ^**^p < 0.01, ^***^p < 0.001, ^****^p < 0.0001, ns, not significant.

Higher PLD and LOX activity could affect the cell membrane, resulting in the occurrence of CI in fruit. As shown in Figures 6A,B, chilling stress rapidly increased PLD and LOX activity in carambola fruit, but GABA-treated fruit exhibited lower PLD and LOX activity during the whole storage. In the control fruit, LOX activity greatly increased from 5 to 15 days at 4°C, and then continuously increased, reaching a peak value of 10383.31 U g^−1^ on 10 + 6 days at 25°C, remained flat from 15 + 2 to 15 + 4 days, and then decreased afterward. In contrast to control, GABA-treated fruit exhibited stable and lower activity of LOX over the storage period (Figure 6B). Also, the PLD activity increased gradually from 799.76 to 909.77 U g^−1^ in the control fruit during storage at 4°C, while the PLD activity in GABA-treated fruit firstly dropped from 799.76 to 682.82 U g^−1^, and then increased on 15 days with a value of 787.26 U g^−1^ still lower than the control. Thereafter, the activity of PLD decreased in both control and GABA-treated fruits when the fruits were transferred to 25°C, but GABA treatment inhibited the increases of PLD activity in treated fruits that were stored anywhere from 10 + 3 to 10 + 9 days and 15 + 2 to 15 + 6 days (Figure 6A).

### Effect of Exogenous GABA on the Content of Endogenous GABA

**Figure 7:**
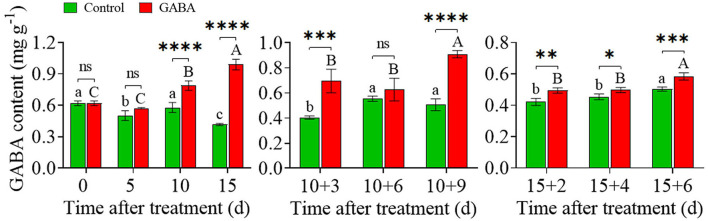
The endogenous γ-aminobutyric acid (GABA) content in carambola fruit during storage at 4 and 25°C. Error bars are expressed as means ± SD (standard deviation) of three replicates (*n* = 3). The asterisks indicate statistically significant differences between treatments at ^*^p < 0.05, ^**^p < 0.01, ^***^p < 0.001, ^****^p < 0.0001, ns, not significant.

GABA accumulated in treated fruit during the whole storage time. The content of GABA increased steadily from.62 to.99 mg g^−1^ in treated fruit compared with the control (0.62 to.41 mg g^−1^) during chilling stress. Also, GABA treatment prevented the decrease and maintained a higher level of endogenous GABA in treated fruit than control from 10 + 3 to 10 + 9 days and 15 + 2 to 15 + 6 days at ambient temperature (Figure 7).

### Fatty Acid Composition

**Figure 8:**
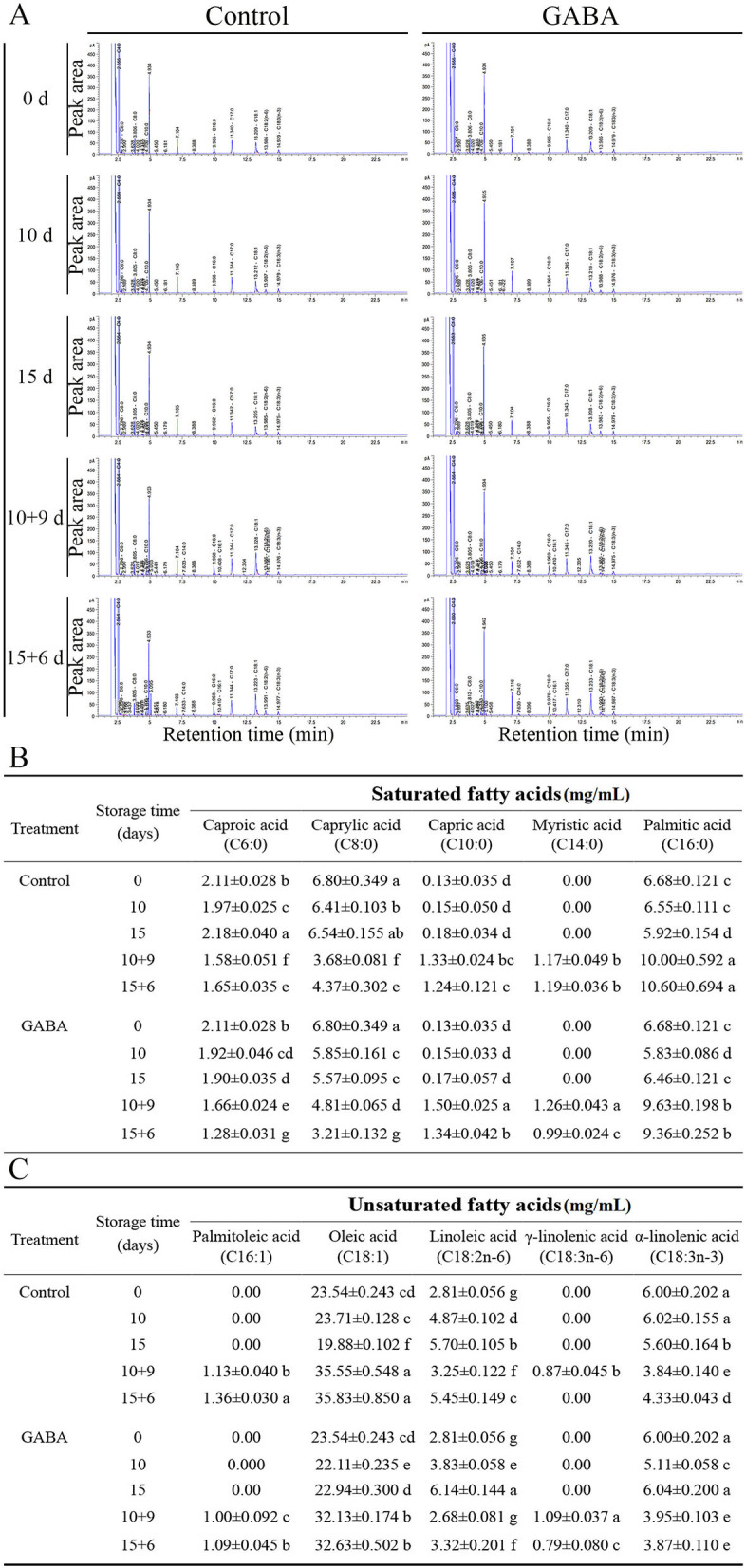
The peak area of fatty acids composition of carambola fruit during storage at 4 and 25°C **(A)**, saturated fatty acids **(B)**, and unsaturated fatty acids **(C)**. Data are means ± SD (standard deviation) of three replicates (*n* = 3).

The changes in fatty acids composition during the entire storage both at 4°C and 25°C are shown in Figure 8. The GC-FID analysis revealed the presence of five major SFAs and UFAs in carambola fruit. The composition of fatty acid was influenced by the application of GABA treatment over storage time. Furthermore, fruit treated with exogenous GABA maintained a lower level of SFA, such as caproic acid (C6:0) and caprylic acid (C8:0) than control fruit, except on 10 + 9 days of storage. Similarly, the level of palmitic acid (C16:0) decreased in treated fruit than control from 0 to 15 + 6 days, except on 15 days of storage. Besides, capric acid (C10:0) and myristic acid (C14:0) exhibited similar decreasing trends but with slight quantitative differences in treated fruit and control (Figure 8B). By contrast, the application of GABA retained a higher level of UFA acids, such as oleic acid (C18:1), linoleic acid (C18:2n6), α-linolenic acid (C18:3n3) in GABA-treated fruit compared with the control only on 15 days of storage at 4°C. Additionally, this treatment inhibited the decreases in γ-linolenic (C18:3n6) in treated fruit only on 10 + 9 days of storage. Simultaneously, it did not affect palmitoleic acid (C16:1) during the whole storage (Figure 8C). Indeed, this increase in UFA (on 15 and 10 + 9 days) and decrease in SFA over storage time might induce cold tolerance in GABA-treated fruit compared with control.

## Discussion

Chilling stress is one of the most threatening abiotic stresses that seriously affect a fruit lifespan. In plants, endogenous GABA accumulation was induced by chilling stress (23). It had been indicated that, within 5 min of cold treatment, GABA concentration climbed up 20–40 folds in soybean leaves (32). Generally, the storage of plants under cold temperature leads to a physiological disorder known as CI. Exogenous GABA confers strong chilling tolerance to plants subjected to cold stress *via* different processes. In recent work, exogenous GABA treatment (1 mM) alleviated chilling symptoms in zucchini fruit at 4°C for 14 days (22), and 5-mM GABA treatment reduced CI in cucumber fruit during storage at 1°C for 5 weeks (10). The present results also showed that 2.5-mM GABA treatment was effective in inhibiting CI symptoms in carambola fruit during storage at 4°C for 15 days and after the fruits were transferred to 25°C, and extended their postharvest shelf life. The respiration rate, weight loss, and flesh firmness are good physiological indicators for evaluating carambola fruit quality, and reflecting the manifestation of CI during storage. After harvest, the carbohydrates in fruit break down into carbon dioxide and water that reflect the respiration activity. The role of GABA in fruit respiration during postharvest remains still unclear. However, it was well-established that the accumulation of GABA in Arabidopsis mutants lacks enzymes for respiration (20), which showed the crucial role of GABA in plant respiration. The major sites of the GABA metabolic pathway include the cytosol and the mitochondrial matrix, which suggest the key relation between GABA, photosynthesis, and plant respiration (33). The interaction between GABA and CO_2_ concentration in postharvest fruit might be the key phenomenon that could shed light on the role of GABA in reducing the respiration activity during cold storage in carambola fruit. Indeed, for decades, reduced O_2_ and/or increased CO_2_ technique has been commercially used to extend the postharvest life and maintain the quality of various freshly harvested fruits (34). Moreover, it is well-known that the cellular respiration rate of fruits depends on temperature and enzymes (35). As the temperature decreases, the rate of respiration decreases, and as the temperature increases, the rate of respiration increases (36). However, the role of GABA treatment in carambola fruit is to slow down the respiration rate, and, compared to the control, the respiration rate in treated fruit decreases at both chilling and non-chilling temperatures. Meanwhile, GABA treatment had a positive effect on reducing the weight loss and firmness peak in carambola fruit exposed to chilling and non-chilling stresses. It is well-known that loss of postharvest fruit firmness and weight is mainly associated with the respiration process during the ripening stage. There is a wide gate open on the pathways that GABA deeply interacts with the respiration on postharvest fruits, and further studies are needed to comprehend how GABA controls the respiration rate of the fruit under both harsh and normal environmental conditions.

As a nonprotein amino acid, GABA is included in the group of compatible solutes, and, during the respiration in postharvest fruit, the disruption of the homeostasis and the significant generation of toxic molecule of oxygen provoked by the variation of the amount of CO_2_ and O_2_ activates the production of osmolytes, such as proline, phenolic compounds, and GABA. These osmoprotectants counteract with the ROS that are responsible for the increase of the lipid peroxidation, EL, DNA damage, and programmed cell death. Therefore, plants under environmental stresses accumulate proline and GABA in large amount to preserve cellular membrane integrity from oxidative stress and increase stress tolerance (37). It is well-documented that proline acts as a ·OH scavenger and O_2_ quencher in the regulation of the NAD^+^/NADH ratio for impeding oxidative damage of plant (22) and also helping in stabilizing the antioxidant machinery. The increase of proline and GABA content after the application of GABA treatment relieved the oxidative damages in carambola fruit caused by chilling stress.

Like proline and GABA, AsA also is an excellent substrate playing a key role in regulating plant redox homeostasis and preventing damages of biomolecules by decreasing lipid peroxidation. The application of GABA significantly increased the AsA content in carambola fruit during chilling and non-chilling stresses, which contribute to the stabilization and protection of plant cell membrane from oxidative damage. This increase of AsA was efficient to maintain the quality of carambola fruit, which is in agreement with Paciolla et al. (38), showing that the reduction of oxidation by the increment of AsA content preserves the fruit color and firmness and prevents spoilage.

**Figure 9:**
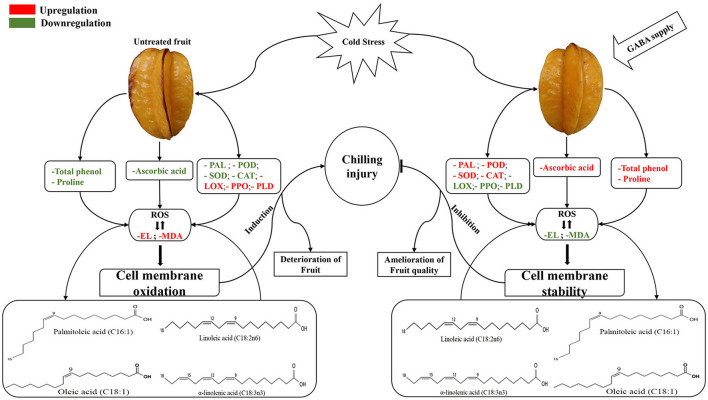
A schematic diagram depicting the ability of GABA modulating enzyme activities and fatty acids composition, and promoting chilling tolerance in carambola fruit.

During respiration and photosynthesis, ROS is naturally produced by plant cells. The overaccumulation of ROS in plants subjected to cold stress is frequently associated with the irreversible cellular compartments damage (mitochondria, chloroplast, and peroxisomes), protein degradation, EL enhancement and lipid peroxidation (9). Plants have evolved diverse adaptative strategies to cope with oxidative stress and ensure their survival, and the common strategy is the enhancement of enzymatic and nonenzymatic antioxidant to maintain cellular redox homeostasis (9). The SOD, CAT, and POD activities were strongly increased by exogenous GABA to eliminate free radicals and maintain cell membrane stability of carambola fruit. For instance, an increase in SOD can convert the dismutation of ${\text{O}}_{2}^{-}$ into H_2_O_2_; thereafter, the generated H_2_O_2_ is catalyzed by CAT and POD to form water and oxygen (11). The increment of these antioxidant enzymes by GABA treatment might participate in delaying lipid peroxidation, EL, and conferring chilling tolerance in carambola fruit. Similarly, GABA treatment upregulated SOD, CAT, and POD in cucumber fruit during cold storage (10) (Figure 9).

PAL activity was involved in the phenolic synthesis through the shikimate/phenylpropanoid pathway (39). This suggested that the total phenol content and PAL activity might be associated with chilling tolerance and the reduction of CI in carambola fruit. In the present work, PAL activity increased in GABA-treated fruit during the storage time. It had been reported that the enhancement of PAL activity in plum fruit improved cell membrane stability, manifested by a reduction of lipid peroxidation and membrane damage measured herein as MDA content and EL, respectively (40). Also, the oxygen-dependent oxidation of phenols to quinones is catalyzed by PPO responsible for tissue browning under abiotic stress, such as chilling stress leading to cellular compartmentalization disruption (39). The present results showed that PPO activity in carambola fruit was effectively inhibited by GABA treatment. The decrease in enzymatic browning of pear fruit was liable for the decrease of PPO activity (41). As an important class of secondary metabolites, phenolic compounds are known to be involved in diverse physiological and metabolic processes in plants (39). Plants accumulate high content of phenolic compounds to protect themselves from damages caused by free radicals-induced oxidative stress (42). Generally, the biosynthesis of total phenolic is triggered in response to abiotic stress, such as chilling stress. The elevation of total phenolic can serve as an indication of plant adaptation to cold storage. However, in the present study, GABA treatment influenced the content of total phenol manifested by the fluctuation during the storage period. These fluctuations might be ascribed to the changes in PAL, PPO, and POD enzyme activities, which synergically affect the changes in total phenol content and cause the fluctuations in total phenol content. It had been indicated that PPO and POD enzymes could catalyze the oxidation of phenolic compounds to quinines by synergistic effect and influence the phenolic metabolism (43) (Figure 9).

The present study aimed to clarify whether the fatty acid composition and antioxidant system in carambola fruit might be involved in the preservation of the membrane stability and fluidity during chilling and ambient conditions. Little studies have reported the variation of UFA and SFA in fruit under harsh environmental conditions and their interactions with GABA. However, a previous study found that a higher UFA increased the membrane fluidity in citrus fruit (17). In the present study, the most UFAs that fluctuated under chilling or ambient temperature were oleic, linoleic, and α-linolenic acids. It was found that the oleic acid (C18:1) is the main synthesized UFA in carambola fruit. Meanwhile, the caproic, caprylic, and palmitic acids were the SFA that fluctuated the most. These striking changes might be related to the oxidative degradation of fatty acids inside the peroxisome. Indeed, fatty acid metabolic flux and lipid peroxidation are crucial elements that maintain the homeostasis, thus preserving the membrane stability to promote fruit storage under both stress and non-stress conditions [(44); 15]. After 21 days of treatment, exogenous GABA enhanced significantly the content of γ-linolenic (C18:3n6) that was one of the interesting differences marked during this experiment. It suggested that γ-linolenic plays a major role in maintaining the membrane stability and fluidity of carambola fruit, and GABA is able to upregulate this UFA when it is necessary. The unsaturation of membrane lipid plays a crucial role in the membranes of fruits by protecting them against oxidative stress. The degradation of membrane lipid affects the fruit membrane structure and function, leading to the development of CI (6). During storage at low temperature, the cell membrane of the fruit undergoes physiological changes, described as softening. Fruit softening is an indicator of the manifestation of CI. The increase in the membrane unsaturation status, due to an increase in UFA, such as linoleic and linolenic acids, maintained the membrane integrity (6), and retarded the occurrence of CI. Besides, linolenic and linoleic acids are the main substrates of LOX enzymes in plant cells. PLD and LOX are two enzymes responsible for the degradation of UFA, and the reduction of membrane integrity of a cell (6). LOX principally catalyzes the oxidation of PFA and esters to form fatty acid hydroperoxides through the addition of the intramolecular oxygen either the 9 or 12 positions (6). Generally, the saturation of fatty acids led to the increase of LOX and PLD activities, which was associated with the occurrence of CI, alteration of membrane lipid composition, and degradation of fruit properties (6). However, in the present study, GABA treatment plays a crucial role by downregulating the activity of LOX and PLD enzymes, thus improving chilling tolerance in carambola fruit, but it reduced the activities of UFA in carambola fruit on 5, 10, 10 + 9, and 15 + 6 days of storage. With further analysis in the present work, it was thought that the application of 2.5-mM GABA was not sufficient in keeping the desired high unsaturation of fatty acid throughout the storage except on 15 days; however, it was efficient in improving antioxidant machinery and physiological characteristics of carambola fruit. In regard to this, Aghdam et al. (26) reported that 5-mM GABA treatment inhibited CI in anthurium cut flowers under chilling temperature by increasing membrane lipid unsaturation and decreasing LOX and PLD activity. Additionally, safranin O/fast green staining revealed *via* microscopic images the damage on carambola fruit cell walls from 5 to 15 + 6 days; and this damage caused by chilling stress became progressively more pronounced from 15 to 15 + 6 days. However, GABA treatment retarded cell walls degradation of carambola peel. This phenomenon might be due to the inhibition of LOX activity and the enhancement of the content of γ-linolenic acid by the application of GABA. Nevertheless, the effect of GABA on fatty acids needs to be more investigated in carambola fruit. Meanwhile, it is very important to notice that, under chilling condition, the increase of the fluctuations of the UFA or SFA was not required for the role of GABA in the regulation of the membrane stability of carambola fruit.

## Conclusion

In conclusion, the present study showed that exogenous GABA efficiently delayed the occurrence of CI and maintained a higher quality of carambola fruit during low temperature. The degree of membrane lipid peroxidation is reduced in GABA-treated fruit than that in control due to the ability of GABA treatment to enhance the antioxidant defense systems and decrease EL and MDA content. Furthermore, the accumulation of proline plays a vital role in inducing acclimation to cold stress. The fluctuation of UFA and SFA in GABA-treated fruit was associated with chilling tolerance in carambola fruit. Overall, exogenous GABA could be a good approach to agroindustry, allowing the fruit to fit cold storage and also facilitating transportation over long distance. However, further work is needed to shed light on the molecular complex of GABA to improve the quality of fruit during storage at low temperature.

## Data Availability Statement

The original contributions presented in the study are included in the article/Supplementary Material, further inquiries can be directed to the corresponding author.

## Author Contributions

FN: writing–original draft. FN and WD: conceptualization and methodology. TC: resources. FN and EC: software, data analysis, investigation, and validation. XX: writing–reviewing and editing, project administration, supervision, and funding acquisition. All the authors have read and approved the published version of the manuscript.

## Funding

This study was supported by the National Natural Science Foundation of China (31872160), and the Open Foundation of Tianjin Key Laboratory of Postharvest Physiology and Storage of Agricultural Products (kf2019002).

## Conflict of Interest

The authors declare that the research was conducted in the absence of any commercial or financial relationships that could be construed as a potential conflict of interest.

## Publisher's Note

All claims expressed in this article are solely those of the authors and do not necessarily represent those of their affiliated organizations, or those of the publisher, the editors and the reviewers. Any product that may be evaluated in this article, or claim that may be made by its manufacturer, is not guaranteed or endorsed by the publisher.
