# Supplementary material for: Alleviation of Postharvest Chilling Injury of Carambola Fruit by γ-aminobutyric Acid: Physiological, Biochemical, and Structural Characterization
Source: Front Nutr. 2021 Nov 16;8:752583. doi: 10.3389/fnut.2021.752583 (PMC8637291; doi:10.3389/fnut.2021.752583)
Supplement: Supplementary file 1 [file Data_Sheet_1.docx]

Supplementary Material

**
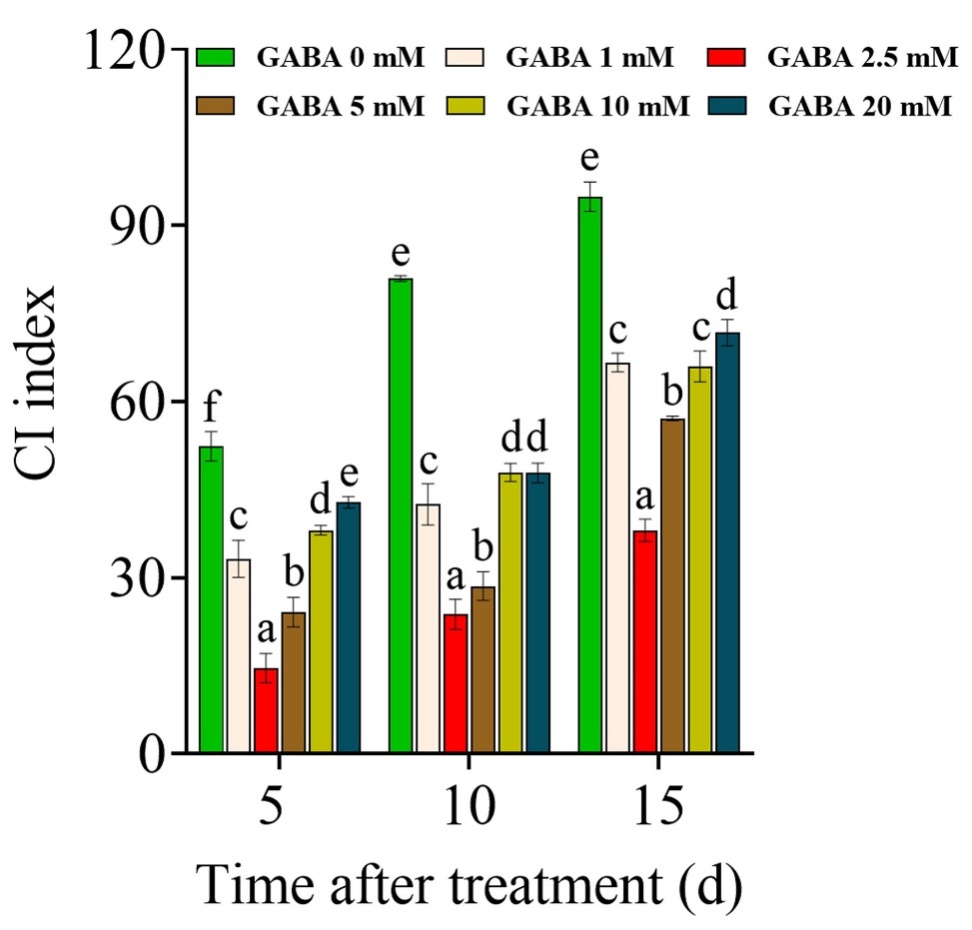
**

**Supplementary Figure 1.** Effect of GABA treatment (0, 1, 2.5, 5, 10 and 20 mM) on CI of carambola fruit during storage at 4 ^o^C for 15 d. The letters above the bars represent significant differences based on Tukey’s multiple comparison test (p < 0.05).

**
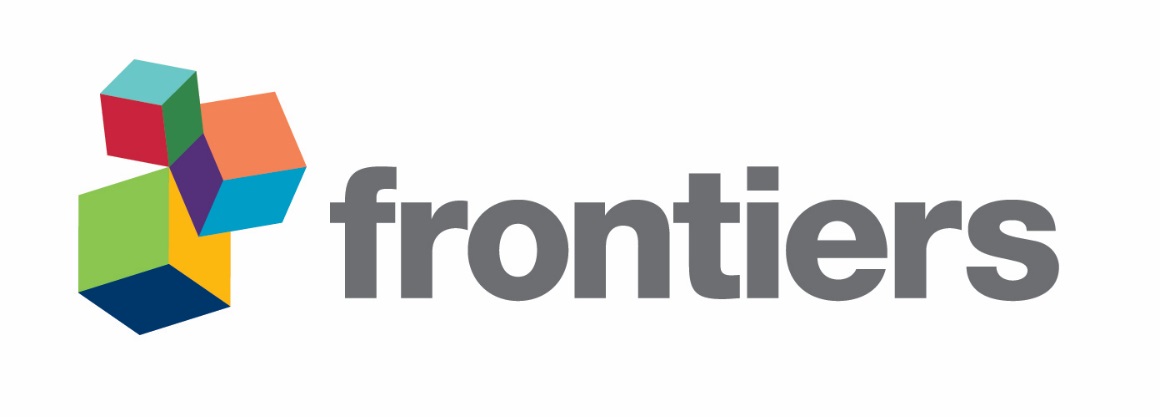
**
